# Uptake of care and treatment amongst a national cohort of HIV positive infants diagnosed at primary care level, South Africa

**DOI:** 10.1186/s12879-019-4342-3

**Published:** 2019-09-16

**Authors:** Elelwani Mathivha, Steve Olorunju, Debra Jackson, Thu-Ha Dinh, Nicolette du Plessis, Ameena Goga

**Affiliations:** 1 Mamelodi Hospital, Pretoria, 0112 South Africa; 2 Department of Paediatrics, University of Pretoria, Pretoria, 0001 South Africa; 3 Biostistics Unit, South African Medical Research Council, Pretoria, 0001 South Africa; 4 Health Section, United Nations Children’s Fund (UNICEF), New York, NY 10017 USA; 5 School of Public Health, University of the Western Cape, Cape Town, 7535 South Africa; 6 Division of Global HIV and Tuberculosis, Center for Global Health, Centers for Disease Control and Prevention, Atlanta, GA 30329 USA; 7 Health Systems Research Unit, South African Medical Research Council, Pretoria, 0001 South Africa; 8 8 HIV Prevention Research Unit, South African Medical Research Counci, Durban, 3630 South Africa

## Abstract

**Background:**

Loss to follow-up after a positive infant HIV diagnosis negates the potential benefits of robust policies recommending immediate triple antiretroviral therapy initiation in HIV positive infants. Whilst the diagnosis and follow-up of HIV positive infants in urban, specialized settings is easier to institutionalize, there is little information about access to care amongst HIV positive children diagnosed at primary health care clinic level. We sought to understand the characteristics of HIV positive children diagnosed with HIV infection at primary health care level, across all provinces of South Africa, their attendance at study-specific exit interviews and their reported uptake of HIV-related care. The latter could serve as a marker of knowledge, access or disclosure.

**Methods:**

Secondary analysis of data gathered about HIV positive children, participating in an HIV-exposed infant national observational cohort study between October 2012 and September 2014, was undertaken. HIV infected children were identified by total nucleic acid polymerase chain reaction using standardized procedures in a nationally accredited central laboratory. Descriptive analyses were conducted on the HIV positive infant population, who were treated as a case series in this analysis. Data from interviews conducted at baseline (six-weeks post-delivery) and on study exit (the first visit following infant HIV positive diagnosis) were analysed.

**Results:**

Of the 2878 HIV exposed infants identified at 6 weeks, 1803 (62.2%), 1709, 1673, 1660, 1680 and 1794 were see at 3, 6, 9, 12, 15 and 18 months respectively. In total, 101 tested HIV positive (67 at 6 weeks, and 34 postnatally). Most (76%) HIV positive infants were born to single mothers with a mean age of 26 years and an education level above grade 7 (76%). Although only 33.7% of pregnancies were planned, 83% of mothers reported receiving antiretroviral drugs to prevent MTCT. Of the 44 mothers with a documented recent CD4 cell count, the median was 346.8 cell/mm^3^. Four mothers (4.0%) self-reported having had TB. Only 59 (58.4%) HIV positive infants returned for an exit interview after their HIV diagnosis; there were no statistically significant differences in baseline characteristics between HIV positive infants who returned for an exit interview and those who did not. Amongst HIV positive infants who returned for an exit interview, only two HIV positive infants (3.4%) were reportedly receiving triple antiretroviral therapy (ART). If we assume that all HIV positive children who did not return for their exit interview received ART, then ART uptake amongst these HIV positive children < 18 months would be 43.6%.

**Conclusions:**

Early ART uptake amongst children aged 15 months and below was low. This raises questions about timely, early paediatric ART uptake amongst HIV positive children diagnosed in primary health care settings. Qualitative work is needed to understand low and delayed paediatric ART uptake in young children, and more work is needed to measure progress with infant ART initiation at primary care level since 2014.

## Introduction

Although there has been a reduction in new HIV infections amongst children aged 0–14, at the end of 2016, approximately 2.1 (1.7–2.6) million children were infected with human immunodeficiency virus (HIV); 90% of them lived in sub-Saharan Africa [1–3]. Although interventions to prevent mother to child transmission of HIV (PMTCT) have successfully reduced new paediatric HIV infections, paediatric HIV has not been eliminated [4]. Without treatment, paediatric HIV is a rapidly progressive disease, with high mortality [5]. Since the introduction of triple antiretroviral therapy (ART), and particularly early ART, infant survival rates have significantly improved [1, 2, 5, 6]; however, the proportion of children accessing treatment remains unacceptably low. [6, 7]. Although ART coverage for HIV positive children aged 0–14 years increased globally from 28% in 2009 to 74% in 2015, and in South Africa from 29% in 2010 to 55% in 2016, ART uptake amongst young children under the age of 2 years is unknown [3, 8, 9]. In resource limited settings, not all HIV exposed children receive timely and appropriate infant HIV diagnostics and referral into care; this compromises early treatment [1, 7]. In South Africa, task shifting, decentralization of HIV treatment and nurse initiated management of antiretroviral therapy (NIMART) have been implemented to scale-up coverage of HIV care. Data demonstrate that NIMART reduces waiting times, loss to follow-up, transport costs and opportunity costs, provides care closer to patients’ homes, and increases retention in care [10–12]. By 2010 management of paediatric HIV infection was included in the South African chart booklet of the Integrated Management of Childhood Illness (IMCI) strategy, and guidelines recommended ART for all HIV positive infants (children less than 1 year); by 2013 ART eligibility criteria expanded to include all HIV positive children under the age of 5 years [13, 14].

This paper describes the characteristics of HIV positive children aged 6 weeks to 18 months, spread across geographical settings nationally in South Africa, their attendance at study-specific primary health care clinic exit interviews and their uptake of HIV-related care. This provides a deeper understanding of access to care for HIV positive children attending routine, non-specialised health services.

## Methods

### Study design

Secondary analysis of data from HIV positive infants was undertaken. The data were drawn from a nationally representative prospective observational cohort study of HIV exposed infants (HEI), followed up between October 2012 and September 2014 to establish 18-month mother to child transmission of HIV (MTCT) and HIV-free survival. This cohort was established from a nationally representative survey conducted at 580 primary health care clinics / community health centres across all nine provinces of South Africa to measure MTCT at 4–8 weeks postpartum. During the survey, multistage probability proportional to size sampling methodology was used to establish a nationally and provincially representative sample. Hospitals were excluded from the sampling frame to reduce the bias posed by sick populations, and because hospitals do not routinely offer immunization; our sample was drawn from 6 week immunization service points, which have > 95% immunisation coverage. All infants receiving their six-week immunization in selected facilities and their mothers/caregivers were recruited, either consecutively (in facilities without long immunization queues) or systematically (in facilities with long immunization queues based on desired sample size for the day and number of eligible mother/infant pairs in the queue). Infant dried blood spot samples were collected from all consented infants (and not only from infants brought by self-reported HIV positive mothers or whose mothers received interventions to prevent MTCT) and tested at the National Institute for Communicable Diseases (NICD), National Health Laboratory Services for HIV antibody to ascertain HIV exposure. Antibody positive infants or those born to self-reported HIV positive mothers were tested for HIV infection using total nucleic acid polymerase chain reaction (TNA PCR). A closed cohort of HIV antibody positive infants (HEI) was established, and all HEI were subsquently seen at 3-monthly intervals from three until 18 months. At each study visit, mothers / primary carrgivers were interviewed and HEI were tested for HIV (TNA PCR). As per national policy, the laboratory returned all infant HIV test results to their clinic of origin within 1 month. Routine clinic (not research) nurses were required to return results to mothers/parents, confirm the diagnosis and initiate paediatric ART. As per national policy, all children with confirmed HIV infection were eligible for ART, regardless of their CD4 cell count. Results at the 6 week time point have been reported elsewhere[15–17]. and details about how the HIV infected population differs from the rest of the cohort are addressed in the overall 18-month MTCT and HIV-free survival paper, which is currently under review. More information about loss to follow-up in the HIV exposed cohort, from which the HIV infected children came, are provided in the paper by Ngandu et.al. in this series. For this paper, given that confirmation of the infant’s HIV status would have been complete within 1 month of the blood-taking visit, we expected that HIV positive infants should have received HIV-related care (including ART) before they were seen at the next visit, which was scheduled 3 months later. The study visit 3 months after an infant HIV positive diagnosis, or as soon as possible to the 3 month post HIV diagnosis time point if the mother could not be contacted at this time point, was the study exit interview for HIV positive infants. This visit aimed to check that the infant was in HIV-related care, as infant HIV diagnosis was the study endpoint.

### Study population

HIV exposed infants enrolled in the follow-up study at 4–8 weeks postpartum, and diagnosed with HIV infection at six-weeks, or at 3, 6, 9, 12 or 15 months were eligible for this analysis. All infant HIV diagnoses were made at primary health care level, as hospitals were excluded from the sampling frame.

### Data collection methods

Maternal and infant baseline information was obtained from the 4–8 week interview. Data on infant birth weight and gestational age was obtained from the infant’s Road to Health booklet (RTHB), a patient-held booklet issued at birth to all infants born in South Africa. Questions on infant medication were asked at every interview, specifically about infant antiretroviral drug use. A food and medication diary was issued to all HEI at the enrolment visit to assist with recall about medicine and feeding. This diary allowed the mother/caregiver to keep a daily log of medicine and food ingested. The data collector reviewed and collected the diary during each interview. All study exit interviews were conducted at primary health care clinics / community health centers until January 2014; thereafter a non-health facility follow-up site, based on mother’s choice, was allowed. If an HIV positive infant missed the scheduled study exit interview they were invited for interviews at all subsequent time points until they attended the study exit interview, or until the infant reached 18 months, whichever came first. Trained research nurses conducted the study exit interview. All study exit interviews were scheduled to coincide with routine child follow-up visits. For mothers who did not return for study exit interviews, data collectors documented the reason for non-return, where possible. Three attempts (telephone calls on three different occasions) were made to contact each mother to return for a study exit interview. Data collectors searched ART clinic records or registers to ascertain whether children with missed study exit interviews were on ART.

### Data analysis

Data were analysed using STATA version 14. As numbers were small, unweighted descriptive analysis was conducted to explore and understand baseline characteristics of HIV infected infants (at 4–8 weeks postpartum) and uptake of study exit interviews and ART. Chi-square tests were used for categorical variables (Fisher’s exact test, if expected cell count< 5; Cochran-Armitage, for ordinal data with one binary variable) and t-tests or Wilcoxon two-sample test for continuous parametric or non-parametric data, respectively. All *p*-values are 2-sided.

### Ethics approval and consent to participate

Ethics approval for this secondary analysis was obtained from the University of Pretoria Ethics Committee. The main study was approved by the South African Medical Research Council Ethics Committee and the Centers for Disease Control and Prevention. All HIV positive infants who were not on ART during the study exit interview were referred to routine services for ART initiation. Although visits were synchronized with routine clinic visits, caregiver-infant pairs received R100 (approximately 8USD) per visit, as an inconvenience allowance to cover their travel and opportunity costs.

## Results

Of the 2878 HIV exposed infants identified at 6 weeks, 1803 (62.6%) were seen at 3 months, 1709, 1673, 1660, 1680 and 1794 were see at 3, 6, 9, 12, 15 and 18 months respectively. In total, 101 tested HIV positive (67 at 6 weeks, and 34 postnatally). HIV positive infants were born at an average gestation of 38 weeks ranging from 24 to 42 weeks with a mean birth weight of 2.8 kg. Of these infants, 18% were born before 37 weeks gestation and 20/101 (19.8%) had birth weights less than 2.5 kg. Most of these 101 HIV positive infants were born to single mothers (76.2%), with a mean age of 26.7 years, grade 8–12 education (75.2%) and poor access to basic amenities. Approximately three-quarters (75.2%) had access to piped water, 50.5% had a pit latrine and 79.2% used electricity as the main source of energy (data not shown). Most caregivers travelled to clinic on foot (57.4%) with an average travelling time of 27 min (range 2–120 min) (data not shown). Socioeconomic circumstances were suboptimal: 24% of caregivers reported cutting the size of their meals due to non-availability of food. Only a third (33.7%) of pregnancies were reported as planned. There was poor integration between mother and infant care and between infant immunisation and HIV care (data not shown). Most mothers (74.3%) reported receiving support (from a health care worker or family member) during pregnancy and delivery (data not shown). Most mothers (62.42%) were not yet on lifelong ART. Of the 44 mothers who knew their CD4 cell count the median was 346.8 cell/mm3. Four of the 101 mothers, (4.0%) self-reported that they had been diagnosed with TB disease by 6 weeks postpartum.

Of the 101 HIV positive infants we were able to trace 59 (57.6%) to conduct a study exit interview (Table 1). Of the HIV positive infants who did not present for study exit interview (*n* = 42), approximately a third (*n* = 13) died between the 6 and 12- month interview, and the rest were difficult to contact (38%), refused further follow up (28%) or had relocated (2%), data not shown. There were no significant differences between HIV positive infants who presented for a study exit interview and those who did not (Table 1). Of the 59 seen for a study exit interview, only 34 (57.6%) were seen 3 months after the infant’s HIV positive diagnosis. The remainder were difficult to trace, and seen at later time points (Table 2). Amongst the 59 children who attended the study exit interview, 88.1% (52/59) were brought by their mothers. Of the infants not brought by mothers, caregivers reported that mothers were alive but not available on the day of the interview. Prior access to HIV-related care amongst infants attending study exit visits, was non-existent or delayed (Table 2); only two caregivers (3.4%) reported that their HIV positive child was on ART.

**Table 1:** Univariate analysis of factors associated with returning for a study exit interview amongst HIV positive infants aged 6 weeks till 18 months, South Africa, 2012–2014

Characteristics	Category	Returned for study exit interview *n* = 59			Did not return for study exit interview *n* = 42			P
		Number	Mean/Proportion	95% CI	Number	Mean/Proportion	95% CI	
Mean Age		59	26.9 ± 5.4^a^	[25.6, 28.3]	41	26.3 ± 6.7^a^	[24.2, 28.4]	0.74
Marital status	Single	45	76.3	[63.5, 85.6]	32	76.2	[60.7, 86.9]	0.99
	Other	14	23.7	[13.9, 35.4]	10	23.8	[12.2, 37.0]	
Meal Cut in size due to poor socio-economic circumstances	Yes	14	23.7	[13.1–39.3]	10	23.8	[13.1, 39.3]	0.99
	No	45	76.3	[63.5, 85.6]	32	76.2	[60.7, 86.9]	
Maternal Education	0 up to Grade 7	18	30.5	[20.0, 43.6]	6	14.6	[6.6, 29.4]	0.06
	Grade 8 and above	41	79.5	[56.4, 80.0]	35	85.4	[70.6,93.4]	
Experienced discrimination due to HIV status	Yes	8	16.3	[8.2, 29.8]	3	9.4	[2.9, 26.1]	0.36
	No	41	83.7	[70.2, 91.8]	29	90.6	[73.9, 97.1]	
Disclosure to friend or family	Yes	41	69.5	[57.4, 80.0]	28	66.7	[50.9, 79.4]	0.77
	No/other	18	30.5	[20.0, 43.6]	14	33.3	[20.6, 49.1]	
Infant hospitalised by 6 weeks for ≥1 nights	Yes	3	5.1	[1.6, 14.9]	2	4.9	[1.2 18.0]	0.96
	No	56	94.9	[85.1, 98.4]	39	95.1	[82.0, 98.8]	
Mother hospitalised between 6 and 14 weeks for ≥1 night	Yes	1	2.6	[0.03, 17.6]	0	0	–	0.32
	No	37	97.4	[82.4, 99.7]	11	100.0	–	
Mother on ART	Yes	25	51.0	[37, 64.9]	13	40.6	[24.8, 58.6]	0.4
	No	40	49	[35.1, 63.0]	19	59.4	[41.4, 75.2]	
Infant had difficulty breathing in last 2 weeks	Yes	6	10.2	[4.6, 21.1]	3	7.3	[2.3, 20.9]	0.62
	No	53	89.8	[78.9, 95.4]	38	92.7	[79.297.7]	
Infant had diarrhoea in last 2 weeks	Yes	1	1.7	[0.2, 1.1]	3	7.3	[2.3, 20.8]	0.21
	No	58	98.3	[88.5, 99.9]	38	92.7	[79.2, 97.7]	
Time travelling to clinic (mins)		**59**	27.8 ± 18.3	[23.0, 32.6]	42	25.6 ± 14.2	[21.2,30.0]	0.50
Infants birth weight (kg)		**59**	2.8 ± 0.53	[2.7, 3.0]	41	2.8 ± 0.55	[2.6, 3.0]	0.98
Infants gestation at birth		**43**	37.9 ± 2.3	[37.2, 38.6]	28	37.9 ± 3.2	[36.6,39.1]	0.79
Infants weight at 6 weeks		**54**	4.4 ± 0.76	[4.21,4.6]	38	4.22 ± 0.9	[3.9,4.5]	0.20

^a^standard deviation

**Table 2:** Timing of infant HIV positive test, timing of study exit interview and access to infant HIV-related care

Timing of study exit interview	Infant HIV positive detected at 6-week (4–8 weeks) HIV test *N* = 67				
	Had a study exit interview, Number, No, (%)	Caregiver reported during study exit interview:			
		Mother interviewed at study exit	Child referred into ART	Visited an ART clinic	Child on ART
3 months	19 (28.3)	15	1	1	1
6 months	7 (10.4)	7	1	1	1
9 months	2 (3.0)	2	0	0	0
12 months	5 (7.5)	5	0	0	0
15 months	3 (4.5)	2	0	0	0
18 months	1 (1.5)	1	0	0	0
Total positives at 6 weeks with study exit interview	37/67 (55.2)	32/37 (86.5%)	2/37 (5.4)	2 (5.4)	2 (5.4)
	Infant became HIV positive between > 8 weeks and 19 weeks, detected at 3 month visit *N* = 18				
	Had a study exit interview, No (%)	Caregiver reported during study exit interview:			
		Mother interviewed at study exit	Child referred into ART	Visited an ART clinic	Child on ART
6 months	7 (38.8)	6	0	0	0
9 months	0	0	0	0	0
12 months	1 (5.6)	1	0	0	0
15 months	1 (5.6)	1	0	0	0
18 months	0	0	0	0	0
Total positives at 3 months with study exit interview	9/18 (50.0%)	8/9 (88.9%)			
	Infant became HIV positive between > 3 and 6 months, detected at 6 month visit N = 6				
	Had a study exit interview, n (%)	Caregiver reported during study exit interview:			
		Mother interviewed at study exit	Child referred into ART	Visited an ART clinic	Child on ART
9 months	2 (33.2)	2	0	0	0
12 months	1 (16.7)	1	0	0	0
15 months	1 (16.7)	1	0	0	0
18 months	1 (16.7)	1	0	0	0
Total positives at 6 months with study exit interview	5/6 (83.3)	5/5 (100%)			
	Infant became HIV positive between > 6 and 9 months, detected at 9 month visit N = 4				
	Had a study exit interview, n (%)	Caregiver reported during study exit interview:			
		Mother interviewed at study exit	Child referred into ART	Visited an ART clinic	Child on ART
12 months	3 (75.0)	2	0	0	0
15 months	0(0)	0	0	0	0
18 months	0(0)	0	0	0	0
Total positives at 9 months with study exit interview	3/4 (75.0)	2/3 (66.7%)	0	0	0
	Infant became HIV positive between > 9 and 12 months, detected at 12 month visit N = 4				
	Had a study exit interview, n (%)	Caregiver reported during study exit interview:			
		Mother interviewed at study exit	Child referred into ART	Visited an ART clinic	Child on ART
15 months	3(75%)	3	0	0	0
18 months	0	0	0	0	0
Total positives at 12 months with study exit interview	3/4 (75%)	3/3 (100%)	0	0	0
	Infant became	HIV positive between > 12 and 15 months, detected at 15 month visit *N* = 2			
	Had a study exit interview, n (%)	Mother interviewed at study exit	Child referred into ART	Visited an ART clinic	Child on ART
Total positives at 15 months with study exit interview at 18 months	2 (100)	2 (100%)	0	0	0
TOTAL	59/101 (58.4%)	52/59 (88.1%)^a^	2 (3.4%)	2 (3.4%)	2 (3.4%)

^a^For the 7 infants not brought by mother, mother was alive but not available on day of interview. 67 infections detected at 6 weeks and 34 detected postnatally

## Discussion

Using data from a nationally representative study to determine MTCT and HIV-free survival at 3, 6, 9, 12, 15 and 18 months, we demonstrate poor uptake of study exit interviews and extremely low ART uptake (3.4%), despite guidelines recommending ART initiation as soon as possible in HIV positive children < 5 years. If we hypothesise 100% ART uptake amongst children who did not attend the study exit interview paediatric ART uptake would have been 43.6% (44 children). This uptake is still sub-optimal. Given that we found no significant differences in baseline characteristics between infants who returned for study exit interview and those who did not, we postulate that 100% ART uptake in the latter group is highly unlikely. Thus, 43.6% is likely to be an over-estimate of early ART uptake. The poor ART uptake may have been because mothers were waiting for the study nurse to counsel and advise them, or were afraid to take their children to the clinic timeously, or that clinics were still waiting for the confirmatory HIV result, or were still in the process of pre-ART counselling and preparation. Delays in receipt of infant HIV test results have been reported in South Africa, [18]. An observational PMTCT cohort study conducted at Rahima Moosa Mother and Child hospital, Johannesburg between August 2008 and December 2010 demonstrated a median delay interval of 10 weeks recorded between a positive six-week HIV PCR test and initiation of ART with median ART initiation age at 16 weeks of age[19, 20]. At the time of our study UNAIDS estimated that paediatric ART coverage was 49% amongst children less than 14 years in South Africa, compared with a more than 90% ART coverage amongst pregnant women.[4] Our results for children less than 18 months of age are significantly below this modelled national estimate either because the modelled estimate looks at overall access to ART amongst children, and not early access in the first 18 months of life, or due to small numbers of HIV positive children in our study.

Since the discussion of these results at national PMTCT Technical working groups, the National Institute for Communicable Diseases implemented a system that sends out weekly reports to each district, to facilitate tracing of HIV. positive children. The impact of this system remains to be determined.

Our work has several limitations. Although this was a large prospective study drawn from a nationally representative sample of HIV exposed infants, recruited from 580 randomly selected primary health care facilities around South Africa, the number of HIV positive children was small, and analysis did not account for survey design. This, together with the small proportion who returned for study exit interview precluded detailed modelling, and may have biased our assessment of ART uptake. Additionally, all data on ART uptake were self-reported and non-uptake could reflect that the infant was not on ART or that the mother/caregiver did not report that the child was on ART. This is unlikely given the rapport and relationship that developed between mothers and data collectors from recruitment at 6 weeks, and the fact that we used food and medication diaries. We were unable to search laboratory databases to ascertain access to ART or viral suppression, as we did not document infant names in the research database, and had no identifier that linked our study number with a laboratory number. Data collectors had no success finding HIV positive children in clinic ART records/registers. Amongst the HIV positive infants who died, we do not know their ART uptake and response to treatment.

## Conclusions

Our results emphasise the need to strengthen primary health care systems for early paediatric HIV-related care in the first 18 months post-delivery. For policy makers, and at programmatic level, we recommend strengthening outreach systems to find and support HIV positive children and their families[21–23], as poor ART knowledge and false perceptions of high ART costs have been identified as barriers to care and treatment in developing countries [2, 7, 10, 24]. Community-level workers could help overcome these barriers. For primary health care facilities, we recommend integrating maternal and child health services to reduce the number of separate visits for each, and investing in regular systematic community-based tracing of HIV positive children [7, 24]. Further quantitative and qualitative research is needed to understand uptake of paediatric ART and reasons for delayed or poor uptake and adherence.

## Data Availability

Data are available from the authors upon request.

## Abbreviations

ART: antiretroviral therapy
HEI: HIV exposed infant
HIV: human immunodeficiency virus
IMCI: Integrated Management of Childhood Illness
MTCT: mother-to-child transmission of HIV
NICD: National Institute for Communicable Diseases
NICD: National Institute of Communicable Diseases
NIMART: nurse initiated management of antiretroviral therapy
PCR: polymerase chain reaction
PMTCT: prevention of mother-to-child transmission of HIV
TNA PCR: total nucleic acid polymerase chain reaction
